# The molecular landscape of premenopausal breast cancer

**DOI:** 10.1186/s13058-015-0618-8

**Published:** 2015-08-07

**Authors:** Serena Liao, Ryan J. Hartmaier, Kandace P. McGuire, Shannon L. Puhalla, Soumya Luthra, Uma R. Chandran, Tianzhou Ma, Rohit Bhargava, Francesmary Modugno, Nancy E. Davidson, Steve Benz, Adrian V. Lee, George C. Tseng, Steffi Oesterreich

**Affiliations:** 1 Department of Biostatistics, University of Pittsburgh, Pittsburgh, PA USA; 2 Department of Pharmacology & Chemical Biology, University of Pittsburgh, Pittsburgh, PA USA; 3 Womens Cancer Research Center, Magee-Womens Research Institute and University of Pittsburgh Cancer Institute, 204 Craft Avenue, Pittsburgh, PA 15213 USA; 4 Department of Surgery University of Pittsburgh Cancer Center UPCI, Pittsburgh, PA USA; 5 Department of Medicine, Division of Hematology/Oncology, Pittsburgh, PA USA; 6 Department of Biomedical Informatics, University of Pittsburgh, Pittsburgh, PA USA; 7 Department of Pathology Magee-Womens Hospital, Pittsburgh, PA USA; 8 Department of Obstetrics, Gynecology and Reproductive Sciences, Division of Gynecologic Oncology, University of Pittsburgh School of Medicine, Pittsburgh, PA USA; 9 Department of Epidemiology, University of Pittsburgh Graduate School of Public Health, Pittsburgh, PA USA; 10 Five3 Genomics, LLC, Santa Cruz, CA USA; 11 Department of Computational Biology, University of Pittsburgh, Pittsburgh, PA USA; 12 Department of Human Genetics, University of Pittsburgh, Pittsburgh, PA USA

## Abstract

**Introduction:**

Breast cancer in premenopausal women (preM) is frequently associated with worse prognosis compared to that in postmenopausal women (postM), and there is evidence that preM estrogen receptor-positive (ER+) tumors may respond poorly to endocrine therapy. There is, however, a paucity of studies characterizing molecular alterations in premenopausal tumors, a potential avenue for personalizing therapy for this group of women.

**Methods:**

Using TCGA and METABRIC databases, we analyzed gene expression, copy number, methylation, somatic mutation, and reverse-phase protein array data in breast cancers from >2,500 preM and postM women.

**Results:**

PreM tumors showed unique gene expression compared to postM tumors, however, this difference was limited to ER+ tumors. ER+ preM tumors showed unique DNA methylation, copy number and somatic mutations. Integrative pathway analysis revealed that preM tumors had elevated integrin/laminin and EGFR signaling, with enrichment for upstream TGFβ-regulation. Finally, preM tumors showed three different gene expression clusters with significantly different outcomes.

**Conclusion:**

Together these data suggest that ER+ preM tumors have distinct molecular characteristics compared to ER+ postM tumors, particularly with respect to integrin/laminin and EGFR signaling, which may represent therapeutic targets in this subgroup of breast cancers.

**Electronic supplementary material:**

The online version of this article (doi:10.1186/s13058-015-0618-8) contains supplementary material, which is available to authorized users.

## Introduction

Though only 7 % of all invasive breast cancers are diagnosed in women <40 years old [1], breast cancer represents the most frequent non-skin cancer (30−40 %) among younger women [2]. Breast cancers in younger women tend to be associated with poorer survival [2–4], and are more often diagnosed at a later stage of the disease [5, 6]. Several retrospective cohort studies have examined differences in clinical biomarkers in premenopausal (preM) and in postmenopausal (postM) tumors. Age has been shown to be an independent risk factor even after correction for stage, treatment, and tumor characteristics [2], and younger women are more likely to develop tumors with less favorable prognostic characteristics [6]. Also, young women with breast cancer are reported to have less favorable histopathological and survival outcomes as compared to elderly women [7].

Genomic and molecular alterations play a significant role in breast cancer biology. The well-known 50-gene subtype predictor, PAM-50, was developed using microarray data to provide prognostic and predictive information [8]. However, studies that address the unique molecular changes in preM and postM by multiple *omic* approaches are limited. The most notable study compared DNA copy number and messenger RNA (mRNA) gene expression data in preM and postM breast cancer and concluded that transcriptomic changes, more than genotypic variation, account for age-associated differences in sporadic breast cancer incidence and prognosis [9]. Anders et al. analyzed microarray data from 784 early-stage breast cancers to discover gene sets able to distinguish breast tumors arising in younger women from tumors of older women [10]. A number of genes were identified with different expression between breast cancers in younger and older women. A subsequent update reported that after adjusting for clinical variables, there were no gene expression differences between the previously defined age groups [11].

To our knowledge, the effect of aging on molecular changes in breast cancer has never been comprehensively examined in multiple omic datasets (gene expression, methylation, somatic mutation, copy number variation (CNV) data, etc.). Recently, the establishment of large databases, which comprehensively characterize large numbers of breast cancers, including The Cancer Genome Atlas (TCGA) and Molecular Taxonomy of Breast Cancer International Consortium (METABRIC), has provided the opportunity to analyze preM breast cancer and shed light on the possibility of personalized treatment. We show here that estrogen receptor-positive (ER+) preM breast cancer is molecularly distinct from ER+ postM breast cancer, including changes in gene expression, methylation, copy number, and somatic mutation patterns. We observed activation of druggable pathways in preM tumors, which might represent unique targets in the treatment of preM breast cancer.

## Methods

### Data description

Two publicly available datasets, TCGA and METABRIC, were used in this study. A detailed description of the data used for this study is presented in Additional file 12: Supplementary data. The number of samples representing ER+ and ER− preM and postM tumors is provided in Table 1 and a schematic overview of the available genomic data utilized is in Additional file 13: Figure S1. The clinical variables of primary interest were ER status, menopausal status, stage, age, and survival (Additional file 1). We used age as a surrogate for menopausal status: the preM and postM groups were defined as patients with age ≤45 and age ≥55 years, respectively. To minimize potential misclassifications and to exclude perimenopausal cases, we did not include patients between 45 and 55 years of age in the analysis. Institutional Review Board approval was obtained from the University of Pittsburgh prior to accessing METABRIC data. No informed consent was required as the data are publically available.

**Table 1:** Sample summary for estrogen receptor-positive/estrogen receptor-negative (ER+/ER−) and premenopausal/postmenopausal (preM/postM) breast cancer

Project	Dataset	Platform	ER+ (number)		ER− (number)	
			preM	postM	preM	postM
TCGA	Gene expression array	AgilentG4502A	69	250	33	54
	RNAseq	IlluminaHiSeq	109	372	37	94
	Methylation array	HumanMethylation450	75	233	21	64
	Somatic mutation	IlluminaGA	110	392	39	101
	Reverse phase protein array	Reverse phase protein array	50	183	25	47
	Copy number variation	Affymetrix Genome-Wide Human SNP Array 6.0	107	388	38	100
METABRIC	Gene expression array	Illumina HT 12 arrays	130	1,113	121	227
	Copy number variation	Affymetrix Genome-Wide Human SNP Array 6.0	130	1,113	121	227

The data were downloaded on 30 March 2013 (see Additional file 12: Supplementary methods). *TCGA* The Cancer Genome Atlas, *METABRICK* Molecular Taxonomy of Breast Cancer International Consortium

### ER status

In TCGA, ER status was primarily defined by the associated clinical immunohistochemical analysis (IHC) results. On examination of the expression data there was strong correlation between ER IHC and ESR1 expression (data not shown). In order to include samples with missing ER IHC information a logistic regression model was built to impute missing ER status (see Additional file 12: Supplementary methods). In METABRIC, both ER status measured by IHC and ER status defined by ESR1 expression were provided, having high positive correlation (phi-coefficient = 0.82). For our studies, we used ER status as defined by mRNA expression. In both datasets, there was a higher proportion of ER+ tumor samples in the postM group compared to the preM group (TCGA: 62 % postM and 14 % preM; METABRIC: 72 % postM and 12 % preM) (Additional file 13: Figure S1).

### Stratified analysis of molecular profiling TCGA data

A detailed description of the stratified analysis of the molecular data is provided in the Additional file 12: Supplementary methods. Briefly, for gene expression we fitted a linear model for each gene using the function *lmFit* from R package *limma* [12]. We then computed moderated *t* statistics by empirical Bayes moderation of the standard errors with *p* values adjusted using the Benjamini-Hochberg (BH) method. For methylation we performed a two-sample *t* test for each probe and controlled for false positive results using a false discovery rate (FDR) of 5 %.

We applied two methods for analysis of CNV data: (1) gene-level CNV data and (2) segment-level CNV data. In the first setting, Fisher’s exact test was used to detect whether CNV was different between ER+ preM and postM groups for each gene, setting the FDR at 5 %. To compare regions of amplification and deletion between ER+ preM and postM groups, CNV data were processed with GISTIC version 2.0.16gp, to detect regions of significant amplification and deletion (*q* value 0.25). We used the same GISTIC parameters used by FIREHOSE. Somatic mutation data in TCGA was analyzed based on whole-exome sequencing results. We analyzed mutation data by applying MutSigCV v.1.4. In the TCGA breast cancer data as of 30 March 2013, RPPA data were available for 142 proteins (or protein posttranslational modifications) in 233 ER+ tumors. We performed a *t* test for each protein, with the FDR set at 5 %.

### Pathway analysis

To identify enriched signaling pathways among differentially expressed genes, we applied Ingenuity pathway analysis (IPA) (Ingenuity Systems, Redwood City, CA, USA, [13]) and DAVID functional analysis [14]. To identify pathways when simultaneously integrating gene expression, CNV, somatic mutation data, and methylation data, we implemented PARADIGM [15], an algorithm that predicts individual tumor pathway activity by factor graph. We estimated inferred pathway levels (IPL) for each entity in superPathway at the individual patient level. We further performed gene set enrichment analysis (GSEA) [16] on IPL predictions to identify the enriched superPathway for preM tumors.

### Unsupervised and semi-supervised clustering on preM ER+ tumors

Methods for unsupervised and semi-supervised clustering are described in detail in Additional file 12: Supplementary methods. For unsupervised clustering, we performed hierarchical clustering, ranking variable genes by interquartile range (IQR) and using the average linkage algorithm and 1 minus Pearson correlation as the distance measure. The clustering was performed using the R function *hclust* and the heatmap was generated using the function *heatmap.3*. To identify robust clusters and assess the stability of the identified clusters, consensus *k*-means and hierarchical clustering was performed, again using the 2,500 most variable genes ranked by IQR. The clustering was performed using R package *ConsensusClusterPlus*. We further applied sparse *k*-means [17] clustering using R package *sparcl*. The 5,000 genes with the largest IQR were selected for input into the sparse *k*-means algorithm, and we selected the number of clusters (*k*) to be 3 and weight summation (*Σ*_*i*_ *w*_*i*_) to be 25. The advantage of sparse *k*-means over hierarchical clustering is that it performs automatic feature selection in the algorithm based on sparsity regularization. In Fig. 4a-c, we applied sparse *k*-means to TCGA, used the selected features to validate in METABRIC and then compared the survival differences across clusters.

The above described unsupervised clustering approach does not consider survival information in the cluster formation for risk prediction. Thus, we applied a semi-supervised approach in the METABRIC study to construct survival-associated clusters for risk prediction of future patients [18]. We fitted a Cox proportional hazard model for each gene and tested for the influence of gene expression on survival outcome. We selected genes whose expression significantly associated with survival (*q* value <0.01 after BH adjustment and absolute coefficient >1). We then used this set of genes to perform the *k*-means algorithm. A 10-fold cross-validation approach was used to avoid overfitting of the data. Additional details of the clustering approach are provided in Additional file 12: Supplementary methods.

## Results

### Differences in gene expression comparing preM and postM breast cancer

**Fig. 1:**
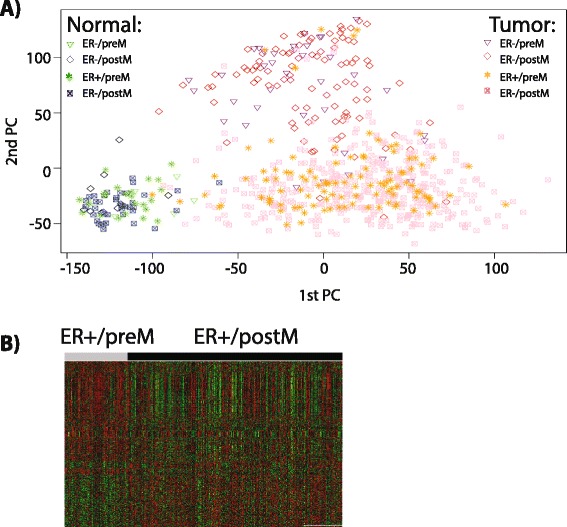
Differences in outcome and gene expression comparing premenopausal (*preM*) and postmenopausal (*postM*) tumors. **a** Principal component analysis (PCA) using RNA seq data from The Cancer Genome Atlas (TCGA). Tumor: *purple triangle*, estrogen receptor-negative (*ER*–)/preM; *red diamond*, ER–/postM; *orange star*, estrogen receptor-positive (*ER*+)/preM; *pink square*, ER+/postM. Normal: *light blue triangle*, ER–/preM; *black diamond*, ER–/postM; *green star*, ER+/preM; *dark blue square*, ER+/postM. **b** Heatmap showing genes differentially expressed (n = 1,609) between ER+/preM and. ER+/postM, using RNA-Seq data. Each gene is normalized to standard normal distribution with *green* indicating lower expression, and *red* indicating higher expression. *PC* principal component

Here we set out to understand differences in molecular make-up between preM and postM breast cancer, starting with the analysis of differential gene expression (DE). As a first step, we performed principal component analysis (PCA) of RNA-seq data (Fig. 1a) and Agilent microarray data (Additional file 13: Figure S2A) from normal and breast cancer samples in TCGA. Normal and tumor samples were separated by the first principal component (PC), while ER+ and ER− tumor samples were separated by the second PC (Fig. 1b, Additional file 13: Figure S2A). PreM and postM patients were not separated by the first two PCs. Further, the third PC did not separate preM and postM patients (data not shown). Similar results were obtained when using methylation data (Additional file 13: Figure S2B).

We used DE analysis to compare gene expression data in preM and postM tumors, stratified by ER status. Analysis was performed using RNA-seq and Agilent microarray data with a focus on RNA-seq data due to the larger number of samples. RNA-seq data were available on 612 preM and postM ER+ tumors and ER– tumors (ER+, number of preM:number of postM = 109:372, and ER–, number of preM:number of postM = 37:94). For ER+ tumors, we found a total of 1,609 and 564 DE genes in RNA-seq data (Fig. 1b) and Agilent array data (Additional file 13: Figure S3A), respectively (gene lists are provided in Additional file 2). The overlap (Additional file 13: Figure S3B) of 301 genes between RNA-seq and Agilent microarray data was statistically significant (*p* <0.0001). Adding a restriction to fold change (>1.5 or <0.5) decreased the number of DE genes to 178 (89.3 % overexpressed in preM) in RNA-Seq data. The top overexpressed genes in preM tumors were *AREG, TFPI2, AMPH, DBX2, RP5-1054A22.3,* and *KLK5*, and the genes with significantly lower expression in preM were *ESR1, CYP4Z1*, and *RANBP3L, FOXD2*, and PEX3. In contrast to ER+ tumors, no DE genes were detected when comparing preM and postM ER– tumors.

Because of the influence of sample size on DE analysis, and the difference in the size of the preM and postM groups, we conducted an analysis using a subsample of ER+ preM and postM tumors of the same size as in the ER– tumors. We again detected a statistically significant number of genes DE between preM and postM in ER+ but not in ER– tumors (data not shown). During the subsampling (n = 100), we identified 28 genes that were consistently detected in more than 10 subsample tests, and 2 genes (AREG and ESR1) were detected as DE in more than 50 subsample tests. Due to the lack of significant differences between ER– preM and postM tumors, our subsequent analyses focused on ER+ disease.

### Differences in methylation, CNV, somatic mutation and proteomics comparing preM and postM ER+ breast cancer

**Fig. 2:**
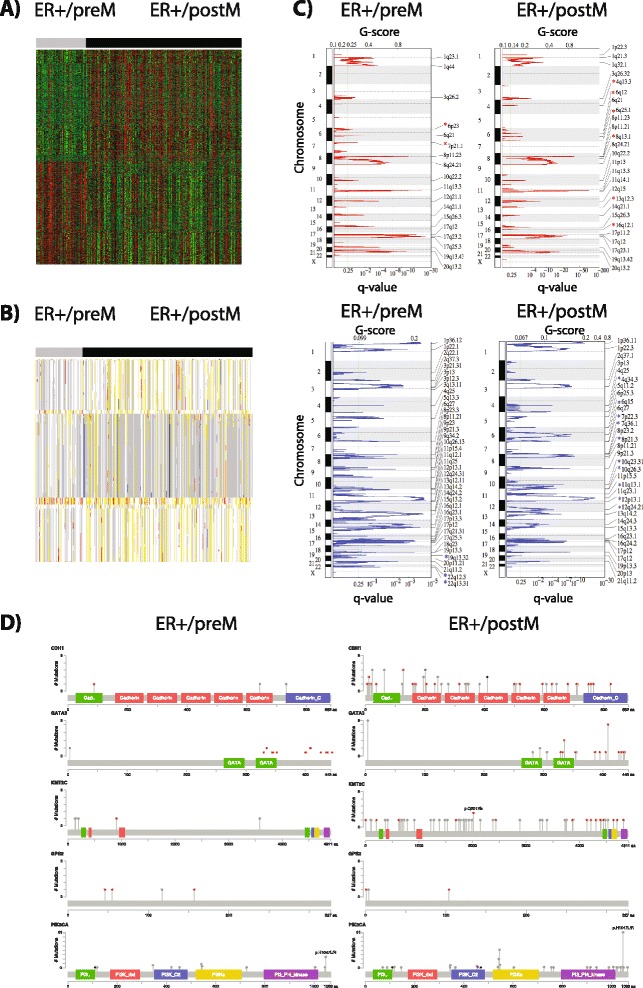
Differences in methylation and copy number variation (CNV) between premenopausal (*preM*) and postmenopausal (*postM*) estrogen receptor-positive (*ER*+) tumors: **a** Heatmap representing significant differences in methylation of 1,738 probes between preM (n = 75) and postM (n = 233) tumors. *Red* and *green* indicate higher and lower levels of methylation, respectively. **b** Heatmap representing significant changes in CNV at gene level between preM and postM ER+ tumors (772 genes). *White* indicates diploid normal copy, *gray* indicates single copy deletion (LOH), *blue* indicates homozygous deletion, *yellow* indicates low-level copy number amplification, and *red* indicates high-level copy number amplification. **c** Peaks of significant amplification (*top panels*) and deletion (*bottom panels*) in preM and postM ER+ tumors. Peaks were identified by GISTIC 2.0. The x-axis represents the *G* score (*top*) and *Q* value (*bottom*). The *vertical green line* represents the significance cutoff *q* value = 0.25. Stars indicate significantly different regions of amplifications or deletions comparing preM and postM ER+ tumors. **d** Mutation distribution in the top five differentially mutated genes (*CDH1, GATA3, MLL3/KMT2C, GPS2, PIK3CA*). *Red*, *black*, and *gray* indicate truncating, in-frame and other mutations, respectively. Figures were generated with cBio MutationMapper [49, 50]

To determine significant differences in methylation between preM and postM ER+ breast tumors, we analyzed methylation data from TCGA. A total of 1,738 probes (mapping to 818 unique genes) were differentially methylated after setting the FDR at 5 % and constraining the absolute difference of the average beta value within the preM and postM groups to be >0.1 (Fig. 2a). Among them, 48 % of the probes (373 genes) were hypo-methylated in preM tumors, while 52 % probes (457 genes) were hyper-methylated in preM tumors. *ESR1, MAT2B, CTSS, DDR2* and *GALNTL2* were the top genes hyper-methylated in preM tumors relative to postM tumors. *RPL3, FBXL16, RASGEF1A, KLF6* and *MCM7* were the top genes hyper-methylated in postM ER+ breast cancer (genes lists are provided in Additional file 2).

We performed a gene-level comparison of CNV between preM and postM ER+ tumors: 772 genes had different CNV, with *SAFB2, TNFSF9, C19ORF70*, and *HSD* having the most significant changes (Fig. 2b) (gene lists are provided in Additional file 2). We also performed GISTIC to identify regions of frequent and significant aberrations in ER+ preM and postM tumors (Fig. 2c). GISTIC identified 26 significant amplifications and 33 deletions in ER+ postM tumors, and 18 significant amplifications and 39 deletions in ER+ preM tumors (Additional file 3). Inspection of the GISTIC score file in Integrated Genomics Viewer (IGV) identified nine regions of deletion (10q26.3, 10q23.31, 11q13.1, 12q24.21, 4q34.3, 6q15, 7p22.3, 7q36.1, 8p21.3) and six regions of amplification (13q12.3, 16q12.1, 4q13.3, 6q25.1, 6q12, 8q13.1) that were unique to ER+ postM tumors. Three regions of deletion (19q13.32, 22q12.3, 22q13.31) and two regions of amplification (6p23, 7p21.1) were uniquely identified in ER+ preM tumors.

To identify genes differentially mutated in preM and postM tumors, we applied the MutSig analysis algorithm. Genes significantly mutated in preM or postM (*q* <0.05) are shown in Additional file 2. Five genes had statistically significantly different mutation rates between preM and postM tumors: *CDH1* (14.5 % vs 2.9 %), *GATA3* (9.4 % vs 18.6 %), *MLL3* (13.3 % vs 4.9 %), *GPS2* (0.8 % vs 3.9 %), and *PI3KCA* (48.7 % vs 37.3 %). The mutation distributions are shown on a diagram of the domain structures of the respective proteins (Fig. 2d). After correction for multiple comparisons only one gene, E-cadherin (*CDH1*) remained differentially mutated between the preM and postM groups. This finding is in agreement with E-cadherin being frequently mutated in invasive lobular carcinoma (ILC), which are more common in older patients. It is possible that additional genes have significantly different mutation rates in preM and postM tumors, but that we failed to detect those due to limited power. Given our sample number of 102 ER+ preM and 384 ER+ postM tumors used for the MutSig analysis, and assuming mutation rates of 0 % and 2.5 %, or 2.5 % and 10 %, we had 81 % and 83 % power to detect those (detailed information on power analysis for our study is shown in Additional file 2).

We determined overall base pair mutation rates, and found significantly increased rates of mutations in postM compared to preM breast cancer (0.99 vs 0.67 mutation/Mb; *p* <0.0001). We asked whether there was a difference in the mutations spectra of ER+ preM and postM tumors and observed an increase in C>T mutations in postM tumors (Additional file 13: Figure S4A) translating to an increase in transitions (Additional file 13: Figure S4B) (preM 43 %, postM 54 %; *p* <0.0001 chi-square test). Further examination of the trinucleotide mutation spectra showed that the increased C>T mutations in postM cancer was limited primarily to the context of a 5′ T and 3′G (TCG>TTG) (Additional file 13: Figure S4C). Additionally, postM cancers were enriched for mutations within TCW motifs that are associated with APOBEC-induced changes (preM 27 %, postM 32 %; *p* <0.001 chi-square test), and this increase was limited to the context of TCT>TAT (Additional file 13: Figure S4D).

To compare protein expression and posttranslational modifications between preM and postM ER+ tumors we studied RPPA data which were available for 142 proteins in 233 ER+ tumors at the time of our data lock. The only significant difference detected was increased expression of ERα in postM ER+ tumors (Additional file 13: Figure S5 and Additional file 2). Phosphorylation of ERα at Ser118 ranked third and was significant at a nominal level (*p* value 0.0016, adjusted *p* value 0.17).

### Identification of signaling pathways enriched in preM ER+ tumors

IPA was used to identify active pathways in preM tumors. We limited the analysis to genes which (1) were differentially expressed between preM and postM ER+ tumors, and (2) were significantly different between preM ER+ tumors and normal tissue using the union of genes identified from both RNA-seq and Agilent microarray data (Additional file 4). IPA analysis revealed integrin signaling as the most significant canonical pathway altered when comparing ER+ preM and ER+ postM tumors (Additional file 5). Pathway enrichment is shown for both RNA-seq and Agilent microarray data analyzed individually (Additional file 13: Figure S6) which showed strong concordance. A potential role for integrin signaling is supported by DAVID functional analysis, which identified focal adhesion as the most significantly altered pathway in preM and postM ER+ tumors (Additional file 13: Figure S7). IPA analysis identified the transforming growth factor (TGF)β pathway as the most significant upstream regulator of the transcriptional network in preM ER+ breast cancer (Additional file 6).

**Fig. 3:**
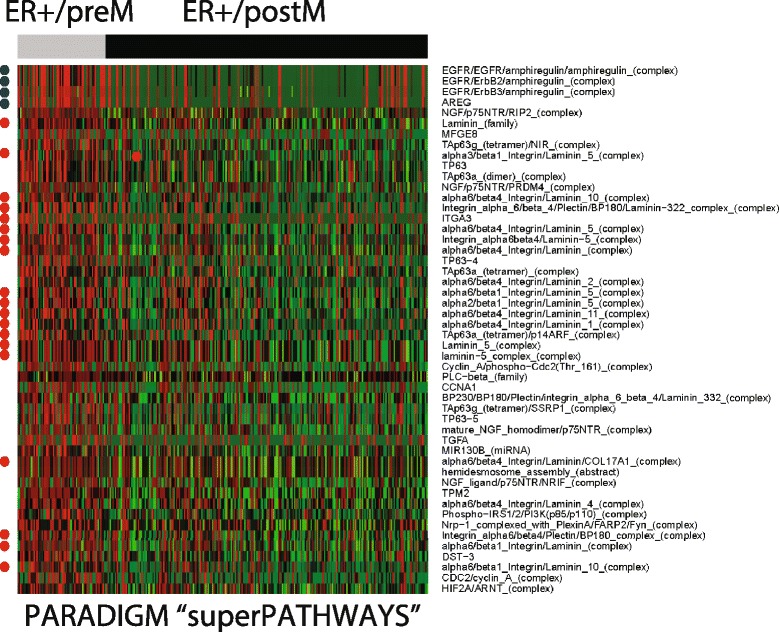
Pathways activated in premenopausal (preM) breast tumors. Top 50 entities in superPathway analysis using Agilent array, copy number variant (CNV) and mutation analysis of The Cancer Genome Atlas (TCGA) data (preM, n = 65 and postM, n = 239). *Green* indicates low activity score and *red* indicates high activity score. *Red stars* refer to pathways related to integrin/laminin signaling, and *blue stars* label EGFR and AREG signaling

In order to integrate the information-rich data from multiple omics platforms we implemented PARADIGM analysis incorporating gene expression, copy number, and methylation changes to infer pathway-level changes. Out of 8,674 entities in the database, 1,026 were significantly different in preM compared to postM tumors (*p* <0.05), with a number of integrin and laminin pathways represented among the top 50 entities (Additional file 13: Figure S8). GSEA [16] on PARADIGM predictions identified enriched superPathways in preM tumors; 65 superPathways were identified as being statistically significant (Fig. 3, Additional file 7), with integrin signaling being the only pathway identified multiple times among them (α6β1 and α6β4 integrin signaling; β1 integrin cell surface interactions; integrin signaling pathway; agrin in postsynaptic differentiation; β5 β6 β7 and β8 integrin cell; surface interactions; α6β4 integrin-ligand interactions; integrins in angiogenesis; integrin cell surface interactions). A two-sample *t* test for 19 integrin/laminin genes showed most significant enrichment of laminins A1, B1, B2, C1, C2, and C3, and integin β4 and α1 (Additional file 13: Figure S9). In addition to the integrin pathway, we took note of the epidermal growth factor receptor (EGFR) pathway, which was significantly activated in preM disease, along with its ligand amphiregulin (AREG) (blue stars in Fig. 3).

### Validation using METABRIC

To validate our findings from TCGA data, we conducted an independent analysis using the METABRIC dataset, containing 130 preM and 1,113 postM ER+ tumors, and 121 preM and 227 postM ER− tumors. An unbiased stratified analysis of gene expression data detected 2,542 differentially expressed genes in ER+ tumors, among which 1,322 genes (52.0 %) were over-expressed in preM ER+ samples (Additional file 8). The same analysis detected only 146 differentially expressed genes in ER− samples, confirming our finding from TCGA data of fewer differences between preM and postM gene expression in ER− tumors. There was a significant overlap between the differentially expressed genes in TCGA and METABRIC; 31 % and 36 % of genes over-expressed in ER+ preM and postM in TCGA, respectively, were also found to be differentially expressed in METABRIC (*p* <0.0001).

We next asked whether integrin signaling was among the top differentially activated pathways comparing preM and postM tumors in METABRIC. We applied IPA and confirmed TGFB1 as the most significantly activated upstream regulator (Additional file 9), and integrin signaling among the top 20 canonical pathways. PARADIGM analysis also identified a number of integrin and agrin signaling among the top 50 differentially activated pathways using GSEA (Additional file 10) (β1 integrin cell surface interactions; α6β4 integrin ligand interactions; integrin cell surface interactions). Also, as in TCGA, EGFR signaling was found to be significantly more active in preM ER+ disease.

### Sub-clusters within ER+ preM tumors with poor outcome

**Fig. 4:**
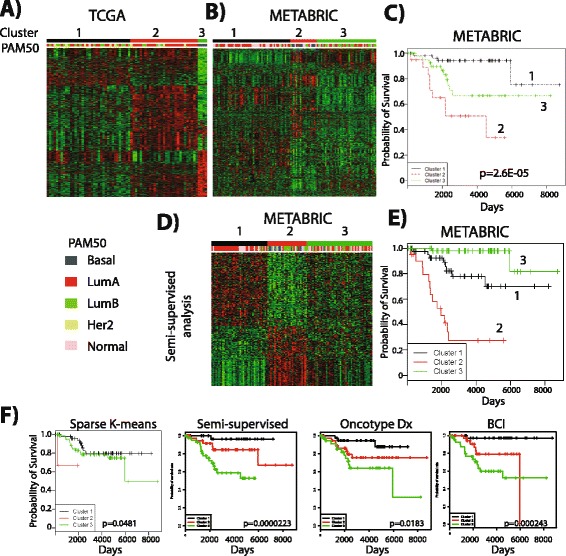
Sub-clusters within premenopausal (*preM*) estrogen receptor-positive (*ER*+) tumors. **a** Clustering result of The Cancer Genome Atlas (*TCGA*) gene expression data by sparse *k*-means yielded three clusters (n_1_ = 36, n_2_ = 29, n_3_ = 4). **b** Sparse *k*-means clustering in Molecular Taxonomy of Breast Cancer International Consortium (*METABRIC*) preM ER+ tumors using genes selected by TCGA (n_1_ = 56, n_2_ = 19, n_3_ = 43). **c** Kaplan-Meier survival curve using clustering result from sparse *k*-means analysis from METABRIC data. **d** Semi-supervised clustering of METABRIC gene expression data from preM ER+ tumor. **e** Kaplan-Meier survival curve of three groups from semi-supervised clustering of gene expression from **d**. **f** Kaplan-Meier survival curves of groups resulting from four different clustering algorithms, with the *p* value from the log-rank test. *Lum* luminal, *Her2* human epidermal growth factor receptor 2

Given the striking molecular differences between ER+ preM and postM tumors (Figs. 1 and 2), we applied several unsupervised and semi-supervised clustering methods to analyze ER+ PreM tumors. Unsupervised hierarchical clustering of ER+ preM tumors from both Agilent and RNA-Seq platforms (Additional file 13: Figure S10) using the top 2,500 differentially expressed genes showed two or more distinct patterns of expression amongst luminal (Lum)A, LumB and human epidermal growth factor receptor 2 (Her2)-like tumors. A small fraction of tumors were classified as basal-like by PAM50, and they clustered together. Sparse *k*-means clustering of ER+ preM TCGA tumor samples with 1,117 selected genes (Additional file 11) identified three clusters. Clusters 1 (n_1_ = 36) and 2 (n_2_ = 29) were mixtures of LumA, LumB and Her2 subtype samples, while cluster 3 (n_3_ = 4) was limited to four basal-like tumors (Fig. 4a). Data were available in METABRIC for 976 genes out of the 1,117 genes detected in TCGA expression (Additional file 11) and we thus used these genes (n = 976), and the same number of clusters (n = 3) for routine *k*-means clustering on ER+ preM tumors in METABRIC (Fig. 4b). As observed in TCGA data (Fig. 4a), such clustering does not simply group the tumors based on the different molecular subtypes as defined by PAM50. Importantly, the three clusters showed very distinct survival in METABRIC (log-rank test *p* value 2.6^E-5^, Fig. 4c), with patients whose tumors were in cluster 1 showing extremely good outcome, and patients whose tumors were in cluster 2 having poor outcomes. The LumA tumors in clusters 1 (n = 33) and 3 (n = 18) had obvious differences in gene expression patterns, and to specifically compare these two subgroups, we examined the difference between them for 6,030 probes that had the largest variation (Additional file 13: Figure S1). After BH adjustment of *p* values, 28 of them remained significant (adjusted *p* value <0.05 and effect size >1 or <−1, Additional file 11). The log-rank test comparing survival between the two LumA groups was significant with a *p* value of 0.003 (Additional file 13: Figure S10).

We next performed semi-supervised machine learning of ER+ preM tumors using the METABRIC dataset: 225 genes were selected following a constraint of absolute effect size >1 and the FDR at 5 %, and *k*-means clustering with *k* = 3 was performed (Additional file 11, Fig. 4d). The three clusters showed distinct survival rates, with patients whose tumors fell into cluster 3 having extremely poor survival (log-rank test *p* = 3^e-11^, Fig. 4e).

Finally, we asked how our clustering approach compared to two widely used multigene assays, OncotypeDx and breast cancer index (BCI) [19, 20] in stratifying risk of recurrence. To avoid overfitting of the data, a 10-fold cross-validation approach was applied. As both Oncotype Dx and BCI classify tumors into three groups (high, intermediate, and low risk) we selected *k* = 3 in all algorithms. Similarly, as OncotypeDx uses 21 genes, we set the number of genes selected in sparse *k*-means and semi-supervised clustering to 21. In sparse *k*-means clustering, the 21 genes were selected according to the weight (largest weight), and in semi-supervised clustering, the 21 genes were selected based on the largest Cox regression analysis score (Additional file 11). Unsupervised sparse *k*-means was the least successful in identifying different outcomes (log rank *p* value 0.0481) (Fig. 4f). Our semi-supervised approach (*p* = 0.000223) and BCI (0.000243) identified three different risk groups, with equally good ability to predict survival. Similar results were obtained for Oncotype Dx that identified three different risk groups (*p* = 0.0183). Additional studies need to be performed using large external datasets in order to validate our clustering approach, and its use to predict outcomes in different preM subgroups.

## Discussion

In this study we used TCGA and METABRIC to identify unique genetic and transcriptomic changes in preM breast cancer compared to postM breast cancer. Differences in gene expression between preM and postM breast cancer were found exclusively in ER+ breast cancer. Integration of multi-omic data analysis identified enrichment of integrin and laminin signaling pathways in preM breast cancer, and TGFβ was identified as the top upstream regulator in both TCGA and METABRIC. In addition, EGFR signaling was activated in preM breast tumors. Semi-supervised clustering using gene expression data from preM ER+ tumors identified three distinct groups of patients with significantly different outcomes.

Using TCGA we only identified significant gene expression differences in ER+ preM and postM breast cancer. None were found in ER− disease. METABRIC also showed only a very minor difference in ER− preM and postM despite containing a much greater number of samples. These findings suggest that the majority of differences between preM and postM breast tumors are driven by altered hormone levels, and thus areonly observed in ER+ disease. Intriguingly, comparing ER+ preM and postM breast cancer, the most altered gene was ESR1 itself, an observation that has previously been reported [10]. PreM breast cancer involved hyper-methylated *ESR1*, lower levels of *ESR1* gene expression, and lower levels of ER protein expression. Conversely, postM ER+ breast cancer involved hypo-methylated ESR1, increased gene expression and increased protein levels. Prior studies have shown an association between ER expression and age or menopausal status [21–25], however, we were unable to find a report on differential *ESR1* methylation comparing preM and postM breast cancer. Intriguingly, one report has shown increased *ESR1* promoter methylation in colon cancer, as a function of age [26]. A preliminary analysis did not reveal significant differences in gene expression of classical ER-target genes between ER+ tumors with hypo-methylated vs hyper-methylated *ESR1* (data not shown), but we plan on performing additional studies, including detailed analyses of different expression, methylation and roles of ER in breast cancer. Our future studies will not only address alteration of ER expression as a function of menopausal status, but also age. This is critical because age is more strongly associated with ER expression than menopausal status in both TCGA and METABRIC (data not shown). Future studies should also address whether there is efficacy of combining epigenetic therapies with endocrine treatment in preM breast cancer patients, possibly using ESR1 methylation as a predictive biomarker.

An intriguing finding is the increased activity of integrin and laminin signaling in preM breast cancer. There are compounds in development targeting this pathway, including volociximab, a chimeric monoclonal antibody that targets integrin α5β1. To date, studies in renal cell carcinoma, pancreatic cancer, malignant melanoma and lung cancer have had promising results [27]. Intetumumab, a monoclonal antibody that targets all members of the αv integrin family, demonstrated increased overall survival when combined with cytotoxic therapy in phase II studies in melanoma [28], but did not improve outcomes in prostate cancer [29].

In addition, the activation of EGFR signaling in preM breast cancer is clearly of potential clinical interest. Other studies have previously identified overexpression of EGFR [10], and its ligand, amphiregulin (AREG), [9] in young breast cancer patients. While therapies targeting EGFR have been studied in breast cancer, these have focused predominately on triple-negative breast cancer, and there have not been previous studies specifically in ER+ patients [30]. The efficacy results have been mixed, and neither overexpression of EGFR by IHC, nor assessment of EGFR pathway analysis microarrays has been an adequate surrogate to predict responsiveness [31–35]. EGFR signaling has been implicated in tamoxifenresistance in preclinical models [36, 37], which could have significant implications for treatment, and lends further credence for EGFR pathway overexpression contributing to worse clinical outcomes in ER+ preM breast cancers. Furthermore, there is evidence for crosstalk between integrin and EGFR signaling in both breast [38] and lung cancer [39], suggesting that successful targeting of these pathways in preM ER+ breast cancer may require a multi-pronged approach.

Our somatic mutation analysis using MutSig identified five genes (*CDH1, GATA3, MLL3, GPS2*, and *PI3KCA*) for which mutation rates were significantly different between preM and postM tumors. After correction for multiple comparisons only one gene (*CDH1*) remained differentially mutated in the preM and postM groups. This is consistent with the fact that mutations in *CDH1* are found almost exclusively in lobular cancers that are enriched in older patients. Interestingly, GATA3, an ER-interacting transcription and chromatin remodeling factor with a role in luminal cell fate and breast tumorigenesis [40–42], was recently shown to be overexpressed in preM breast cancer, and high expression of GATA3 was significantly associated with improved survival in preM women, but not in postM women [43]. Collectively, these data suggest a menopausal status-dependent role for GATA3 in breast cancer.

As expected, we did find lower overall mutation rates in preM compared to postM cancer. Increase of mutation rates with age is likely a general effect of oxidative damage during aging rather than an endocrine response as a result of menopause. Interestingly, further analysis of the mutation spectra showed that postM cancers were enriched for C>T mutations in the context of a 5′ T and 3′G (TCG>TTG), and mutations within TCW motifs that are associated with APOBEC-induced changes. The latter changes were limited within the context of TCT>TAT, an APOBEC motif not typically seen in breast cancer [44]. Together, the increase in these two mutation types matches signature 10 from a recent characterization of trinucleotide mutation context [45]. This signature is thought to be related to defects in *POLE* and DNA mismatch repair genes [44, 46] and thus suggests that defects in *POLE* and DNA mismatch repair genes may play a larger role is postM breast cancers than preM.

In the analysis of gene expression data from TCGA, we combined the differentially expressed genes detected in Agilent array and RNA-Seq data. While platform differences between microarray and sequencing data has been a controversial topic [47, 48], we found relatively good concordance $\left(\overline{r}=0.70\right)$ when examining both platforms performed on the same tumor. Indeed, when we conducted differential analysis for the two datasets using the same set of samples, we identified similar pathways to be activated, with small differences in order/significance level.

Semi-supervised machine learning of preM ER+ patients revealed three groups with strikingly different outcomes. In part, this is expected because we have used the survival information when training the classifier. Unfortunately we are unaware of another large dataset of preM breast cancer with gene expression and outcome data to validate this finding. To avoid overfitting of the data, and provide a more fair comparison, we performed a cross-validation approach, and the results suggest that the semi-supervised machine learning and BCI are equally good predictors, that seem to outperform Oncotype Dx. However, additional studies are necessary, and further research specifically on preM breast cancer will hopefully identify prognostic tests specific for this type of breast cancer and will ultimately lead to personalized therapies.

## Conclusions

In summary, we have demonstrated unique genomic signatures that can differentiate preM ER+ breast cancer from postM ER+ breast cancer. They suggest potential therapeutic strategies such as co-targeting of the laminin/integrin and the EGFR pathways, in addition to anti-estrogen therapies, that should be studied.

## Additional files

Additional file 1:
**The Cancer Genome Atlas (TCGA) and Molecular Taxonomy of Breast Cancer International Consortium (METABRIC) - clinical data summary.** Descriptive statistics of important clinical variables in TCGA and METABRIC data including disease stage, histologic type, estrogen receptor (ER) status, progesterone receptor (PR) status, and human epidermal growth factor receptor 2 (Her2) status. (XLSX 24 kb)
Additional file 2:
**Stratified analysis TCGA.** **Table S1** and **Table S2**: Gene expression-Agilent (ER-pos) (**Table S1**) and RNA-Seq (ER-pos) (**Table S2**). Output from R package “limma” for ER+ tumors. **Table S3**: Methylation (ER-pos). **Table S4**: Copy Number Variation (ER-pos). **Table S5**: RPPA (ER-pos). **Table S6**: Somatic Mutation (ER-pos) (MutSig). **Table S7**: Power Analysis for comparison of two mutation rates with fixed sample size (n1 = 102, n2 = 384), and alpha = 0.05. **Table S8**: Gene expression-Agilent (ER-neg). Output from R package “limma” for ER- tumors. No genes were significant when FDR was controlled at 5 %. **Table S9**: Gene expression-RNA-Seq(ER-neg). Output from R package “limma” for ER- tumors. No genes were significant when FDR was controlled at 5 %. For all tabs, genes highlighted in yellow are significant when FDR is controlled at 5 %. For methylation analysis, there was additional requirement for absolute difference between average beta-value of two groups to be > 0.1. (XLSX 25246 kb)
Additional file 3:
**Copy number variation (CNV) GISTIC The Cancer Genome Atlas (TCGA).** List of significant (*q* <0.25) amplification and deletion peaks identified by GISTIC 2.0, uniquely in premenopausal (preM) and postmenopausal (postM) groups. The columns correspond to the sample group in which the peak is uniquely identified (preM or postM), type of peak (amplification or deletion), cytoband, *q* value, residual *q* value, wide peak boundaries, and number of genes in the region, followed by the list of genes falling under the wide peak boundaries. (XLSX 73 kb)
Additional file 4:
**Stratified gene expression analysis comparing estrogen-receptor-positive (ER+) premenopausal (preM) and Normal groups in The Cancer Genome Atlas (TCGA).** **Table S1.** Gene expression-Agilent. The five columns correspond to gene symbol, log2 fold change, *t* statistics, raw *p* value and adjusted *p* value. Genes (n = 11,366) highlighted in yellow were significant when the false discovery rate (FDR) was controlled at 5 %. **Table S2.** Gene expression- RNA-Seq. The five columns correspond to gene symbol, log2 fold change, *t* statistics, raw *p* value and adjusted *p* value. Genes (n = 14478) highlighted in yellow were significant when the FDR was controlled at 5 %. (XLSX 2881 kb)
Additional file 5:
**Ingenuity pathway analysis (IPA) in The Cancer Genome Atlas (TCGA) union of RNA seq and Agilent data.** Top canonical pathways detected by IPA using the union of differentially expressed genes in Agilent and RNA-Seq expression platforms in TCGA. Four columns correspond to Ingenuity canonical pathway names, −log(*p* value), percentage of genes detected in this pathway, and molecules in this pathway. (XLS 15 kb)
Additional file 6:
**The Cancer Genome Atlas (TCGA) Ingenuity pathway analysis (IPA) upstream regulators.** **Table S1.**, **Table S2.** Top upstream regulators detected by IPA using the union of differentially expressed genes in Agilent and RNA-Seq expression platforms in TCGA. **Table S2.** Top upstream regulators detected by IPA using the differentially expressed gene list from Agilent expression array. Four columns correspond to Ingenuity canonical pathway names, −log(*p* value), percentage of genes detected in this pathway, and molecules in this pathway. **Table S3.** Top upstream regulators detected by IPA using the differentially expressed gene list from RNA-Seq expression array. Four columns correspond to Ingenuity canonical pathway names, −log(*p* value), percentage of genes detected in this pathway, and molecules in this pathway. (XLS 177 kb)
Additional file 7:
**PARADIGM analysis in The Cancer Genome Atlas (TCGA).** **Table S1.** Pathways detected by gene set enrichment analysis (GSEA) with input of gene expression and copy number variation data for the PARADIGM algorithm. The nine columns correspond to the pathway name, size of the pathway, Enrichment score (ES) score, Normalized enrichment score (NES) score, nominal *p* value, false discovery rate (FDR) *q* value, Family-wise error rate (FWER) *p* value, and leading edge (typical GSEA output). **Table S2.** Pathways detected by GSEA with input of gene expression, copy number variation and methylation data for the PARADIGM algorithm. The nine columns correspond to the pathway name, the size of pathway, ES score, NES score, nominal *p* value, FDR *q* value, FWER *p* value, and leading edge (typical GSEA output). (XLSX 88 kb)
Additional file 8:
**Stratified gene expression analysis in the Molecular Taxonomy of Breast Cancer International Consortium (METABRIC).** Gene expression-Illumina. Output from R package *limma*. The five columns correspond to the gene symbol, log2 fold change, *t* statistics, raw *p* value and adjusted *p* value. Genes (n = 2542) highlighted in yellow were significant when the false discovery rate (FDR) was controlled at 5 %. (XLSX 1323 kb)
Additional file 9:
**Molecular Taxonomy of Breast Cancer International Consortium (METABRIC) Ingenuity pathway analysis (IPA) upstream regulators.** Top canonical upstream regulators detected by IPA using the list of differentially expressed genes in METABRIC. Four columns correspond to the Ingenuity canonical pathway names, −log(*p* value), percentage of genes detected in this pathway, and molecules in this pathway. (XLSX 35 kb)
Additional file 10:
**PARADIGM analysis in Molecular Taxonomy of Breast Cancer International Consortium (METABRIC).** Pathways detected by gene set enrichment analysis (GSEA) with input of gene expression and copy number variation data for the PARADIGM algorithm. The nine columns correspond to the pathway name, size of the athway, ES score, NES score, nominal *p* value, false discovery rate (FDR) *q* value, FWER *p* value and leading edge (typical GSEA output). (XLSX 62 kb)
Additional file 11:
**Gene list for clustering premenopausal (preM) estrogen receptor-positive (ER+) tumors.** **Table S1.** Gene list selected by sparse *k*-means algorithm in The Cancer Genome Atlas (TCGA) data. **Table S2.** Genes selected based on TCGA data that are also in the Molecular Taxonomy of Breast Cancer International Consortium (METABRIC) data for validation. **Table S3.** Fixed number of genes (n = 21), gene list being selected from sparse *k*-means in TCGA. **Table S4.** Gene list selected by semi-supervised algorithm in METABRIC. **Table S5.** Fixed number of genes (n = 21), gene list being selected by semi-supervised algorithm in TCGA. **Table S6.** Genes (n = 28) in the LumA cluster that are significantly different between clusters 1 and 3. (XLSX 37 kb)
Additional file 12:
**Supplementary methods.** This file contains additional details of the analyses. (DOCX 36 kb)
Additional file 13: Figure S1. Venn diagram representing datasets from The Cancer Genome Atlas (TCGA) and Molecular Taxonomy of Breast Cancer International Consortium (METABRIC). **Figure S2.** Principle component analysis (PCA) of (A) Agilent array and (B) methylation data. **Figure S3.** Differentially expressed (DE) genes between premenopausal (preM) and postmenopausal (postM) estrogen receptor-positive (ER+) tumors. **Figure S4.** Mutation spectra comparing somatic mutations identified in preM and postM ER+ tumors using MutSig. **Figure S5.** Differences in protein expression between preM and postM ER tumors (RPPA). **Figure S6.** Top canonical pathways enriched in preM ER+ tumors in TCGA RNA-Seq and TCGA Agilent. **Figure S7.** Top pathways identified in DAVID. **Figure S8.** Heatmap for top 50 entities in PARADIGM analysis when integrating Agilent array, copy number variation (CNV), somatic mutation and methylation data. **Figure S9.** Comparison of expression of laminin and integrin genes between preM and postM ER+ tumors. **Figure S10.** Hierarchical clustering of ER+ preM patients on the top 2,500 variable genes: (a) Agilent array; (b) RNA-Seq. **Figure S11.** LumA sub-cluster. (DOCX 3009 kb)

## Abbreviations

AREG: amphiregulin
BCI: Breast Cancer Index
BH: Benjamini-Hochberg
CNV: copy number variation
DE: differentially expressed
EGFR: epidermal growth factor receptor
ER: estrogen receptor
FDR: false discovery rate
GSEA: gene set enrichment analysis
Her2: human epideral growth factor receptor 2
IHC: immunohistochemistry
IPA: Ingenuity pathway analysis
IPL: inferred pathway levels
Lum: luminal
METABRIC: Molecular Taxonomy of Breast Cancer International Consortium
mRNA: messenger RNA
PCA: principle component analysis
postM: postmenopausal
preM: premenopausal
PR: progesterone receptor
TCGA: The Cancer Genome Atlas
TGF: transforming growth factor
