# Supplementary material for: The molecular landscape of premenopausal breast cancer
Source: Breast Cancer Res. 2015 Aug 7;17:104. doi: 10.1186/s13058-015-0618-8 (PMC4531812; doi:10.1186/s13058-015-0618-8)
Supplement: Additional file 12: — Supplementary methods. This file contains additional details of the analyses. (DOCX 36 kb) [file 13058_2015_618_MOESM12_ESM.docx]

**Supplementary Methods**

**Data Description**

Two publicly available datasets, TCGA and METABRIC, were used. Preprocessed level 3 TCGA breast cancer datasets were downloaded from the data portal <https://tcga-data.nci.nih.gov/tcga/tcgaHome2.jsp> by the method of “Data Matrix” on March 30, 2013, including clinical data, gene expression array (AgilentG4502A), RNA-Seq (IlluminaHiSeq), methylation (HumanMethylation450), somatic mutation (IlluminaGA), protein expression (Reverse Phase Protein Array, RPPA) and copy number variation (CNV, Affymetrix Genome-Wide Human SNP Array 6.0). METABRIC data were downloaded from Synapse on July 24, 2013 including gene expression data (Illumina HT 12 arrays) and CNV data (Affymetric SNP 6.0 chip). A schematic overview of the data is presented in Fig S1 and summarized in Table 1. The clinical variables of primary interest were estrogen receptor (ER) status, menopausal status, stage, age, and survival (in days). They are summarized in File S1. In the TCGA data set, there was no significant association between stage and menopausal status. Human subjects exempt IRB approval from the University of Pittsburgh was obtained prior to accessing METABRIC data.

**Imputation of ER status**

In TCGA data, ER status is defined based on overall result of Estrogen Receptor (ER) testing and is highly associated with ESR1 gene expression by fitting a logistic regression model. By means of fitting generalized regression model, we imputed missing/unknown ER status. The fitted model is: TCGA: $\log\left( \frac{p(ER+)}{1-p(ER+)} \right)=0.14+0.89*ESR1$ (p-value < 2e-16). If $0.14+0.89*ESR1$>0, imputed ER status is ER+, otherwise ER-. ER statuses of 5 patients were imputed. TCGA ID for the five patients are: TCGA-A7-A0CH, TCGA-B6-A0I2, TCGA-B6-A0I9, TCGA-C8-A12K and TCGA-C8-A12Y. They are all imputed to be ER+. In METABRIC data, both ER status measured by IHC and ER status defined by ESR1 expression are provided. We used ER status by mRNA expression since this data was complete. Using Agilent microarray data, the numbers of ER+ tumors and ER- tumors in each group were N_preM_ : N_postM_ =69:250 and N_preM_ : N_postM_ =33:54, respectively. Using RNA seq data, the numbers of ER+ tumors and ER- tumors in each group were N_preM_ : N_postM_ = 109:372 and N_preM_ : N_postM_ = 37:94, respectively.

**Preprocessing of methylation and gene expression data**

In all datasets, we first filtered out genes/probes with more than 50% missing values. In the TCGA dataset, gene expression analysis was performed using Agilent array and RNA-Seq. 438 samples are shared by the two datasets, including 390 tumor samples. There were 17,814 genes in Agilent array and 20,502 genes in RNAseq data. Agilent array was originally in log2 scale and RNA-Seq data was log2 transformed. To determine technical concordance between the two platforms, we calculated the pearson correlation of expression values for 16146 genes that were measured by both Agilent and RNA-Seq on the same tumors. After controlling for multiple comparison using Benjamini Hochberg method [[13](#_ENREF_13)], 12251 out of 16146 genes had pearson correlation > 0.7. 15062 genes have significant q-value when controlling FDR at 1% (Fig. S5), showing generally high concordance between Agilent and RNA-Seq data.

For both datasets, we computed mean expression and standard deviation for each gene. We then filtered out genes with small standard deviation (Agilent<0.4; RNA-seq<0.5) and small mean expression (Agilent<0; RNA-Seq<5) to reduce the number of false positives. In Agilent array, after filtering, 8,083 genes remained for ER+ tumor samples and 8,353 genes for ER- tumor samples. In RNA-Seq data, after filtering, 12,309 genes remained for ER+ tumor samples and 13,058 genes for ER- tumor samples. The METABRIC gene expression data set contained 19,602 unique genes, each with multiple probes mapped. For each gene, we selected the probe with the largest inter-Quartile-Range (IQR), and then filtered out genes with mean expression smaller than 5 or standard deviation smaller than 0.3.

The TCGA methylation data contained 485,577 probes. We first removed probes with more than 50% samples missing and then filtered out probes with low IQR (IQR<0.1). No filtering was implemented for CNV and somatic mutation data.

**Stratified analysis of molecular profiling TCGA data**

A detailed description of the stratified analysis of molecular data is provided in the Supplmementary Data. Briefly, for gene expression we fitted a linear model for each gene using the function “lmFit” from R package “limma” [[14](#_ENREF_14)]. Given the fitted model, we then computed moderated t-statistics by empirical Bayes moderation of the standard errors. P-values were adjusted using the Benjamini-Hochberg method. Genes with adjusted p-value < 0.05 were declared as differentially expressed.

For the methylation data, the beta-value was calculated by:

$beta-value=\frac{methylated intensity}{methylated intensity+unmethylated intensity+\alpha}$ ($\alpha$ is a small positive constant),

and was used to quantify the proportion of methylation for each CpG site. We performed a two sample t-test for each probe and controlled for false positive results using a false discovery rate (FDR) of 5%.

We applied two methods for analysis of CNV data: (1) gene-level CNV data (24,174 genes) and (2) segment level CNV data. In the first approach, we downloaded gene level copy number data from UCSC Cancer Browser (version:9/24/2013) generated by the FIREHOSE pipeline (https://genome-cancer.ucsc.edu/proj/site/hgHeatmap/) using the GISTIC2 algorithm. These data are partitioned as follows: homozygous deletion, single copy deletion, diploid normal copy, low-level copy number amplification, and high-level copy number amplification. Fisher’s exact test was used to detect whether CNV was different between ER+ preM and postM groups for each gene, setting FDR at 5%. To compare regions of amplification and deletion between ER+ preM and postM, level 3 Affymetrix SNP 6.0 segmented data (minus_germline_cnv_hg19) was downloaded from FIREHOSE and processed with GISTIC version 2.0.16gp to detect regions of significant amplification and deletion (Q-value 0.25). We used the same GISTIC parameters used by FIREHOSE.

Somatic mutation data in TCGA was analyzed based on whole-exome sequencing results, using MutSigCV v.1.4. Due to some limitations in full functionality of the software when using older version of MAF (as those from our data freeze), we uploaded TCGA exome sequencing files versions_2.1.1.0 (n=102 ER+ preM, and n=384 ER+ postM). We analyzed mutation data by comparing the rate of mutation in preM and postM groups using fisher’s exact test.

In the TCGA breast cancer data as of March 30th, 2013, RPPA data was available for 142 proteins (or protein posttranslational modifications) in 233 ER+ tumors. We performed a t-test for each protein, with an FDR at 5%.

**Remove genes expresses similarly between normal and ER+ tumor in IPA**

The gene lists we come up with from DE analysis may also include genes which show similar expression pattern between preM ER+ tumor samples and normal samples. To identify pathway enriched in preM ER+ samples distinctly in tumor tissues, we need to remove this subset of genes.

Before that, we first performed an analysis to compare the subgroups in adjacent normal samples to see if we can combine them to compare with preM ER+ tumors to increase detection power. Specifically, we performed the following comparisons in adjacent normal samples: (1) ER+ preM vs. ER+ postM; (2) ER- preM vs. ER- postM, for both Agilent and RNAseq data. Similar preprocessing procedure was applied as described in Method section. We applied “limma” to each subset of data afterwards and controlled FDR at 5%. In Agilent data, we detected 1 DE gene in both (1) and 0 in (2). In RNAseq data, we detected 175 in (1) and 0 in (2). After combining preM and postM together in ER+ and ER- respectively, we further compared between ER+ and ER- normal samples in both platforms, and no statistically significant genes were detected.

Later, we combined all adjacent normal samples (N_normal_=49 in Agilent; N_normal_=78 in RNAseq) to compare with ER+ preM tumor group. We first filtered Agilent and RNAseq data for the subgroup of patients (normal and ER+ preM tumors) using the previous criteria and perform “limma” to detect DE genes which control FDR at 5%. Consequently, Agilent data had 8297 genes, with 5503 of them being significant. And RNAseq had 9183 genes being statistically significant from a total of 11991 genes after filtering. We called this DE gene list “DE(Normal vs. ER+/preM)”.

After intersecting the DE(Normal vs. ER+/preM) genes with DE(ER+/preM vs. ER+/postM), there were 380 genes in Agilent data, and 1212 genes in RNAseq data. There are 188 genes overlapping between Agilent and RNAseq.

**Unsupervised and semi-supervised clustering on preM ER+ tumors**

Un-supervised and semi-supervised clustering methods which incorporate survival outcome are frequently used to identify novel patient subgroups for tailored therapy and monitoring [[17](#_ENREF_17), [18](#_ENREF_18)]. To identify distinct sub-clusters in ER+ preM tumors, unsupervised hierarchical clustering was performed on the Agilent and RNA-Seq expression dataset using the 2,500 most variable genes ranked by IQR. We used average linkage algorithm and 1 minus Pearson correlation as the distance measure for the clustering. The clustering was performed using the R function hclust and the heatmap was generated using function “heatmap.3”. To identify robust clusters and assess the stability of the identified clusters, consensus k-means and hierarchical clustering was performed, again using the 2,500 most variable genes ranked by IQR The clustering was performed using R package *ConsensusClusterPlus*.

Sparse K-means was proposed by Witten and Tibshirani [[19](#_ENREF_19)] in clustering observations using a large number of features, due to its efficiency in selecting important genes by imposing sparsity in the target function. In our analysis, 69 ER+ preM solid tumor samples were extracted from Agilent array dataset of TCGA, and 5000 genes with largest IQR were selected for input of sparse k-means algorithm. We selected number of clusters (k) to be 3 and weight summation ($\sum_{i} w_{i}$) to be 25. The genes selected genes in TCGA were then applied to METABRIC in order to test whether the clusters were associated with survival time by log-rank test. The clustering was performed using R package *sparcl*.

Another approach for clustering of preM tumors is to use survival data in a feature selection procedure [[20](#_ENREF_20)]. Specifically, after preprocessing, we fitted cox proportional hazard model for each gene on survival time, and test for the influence of gene expression on survival time. We selected genes whose expression could significantly predict survival (q-value <0.01 after BH adjustment and absolute coefficient >1). We then used this set of genes to perform k-means algorithm. A 10-fold cross-validation was applied to the semi-supervised approach to avoid overfitting. Therefore, the patients were randomly partitioned into 10 equal-sized folds(8 groups with 12 patients in each fold and 2 groups with 11 patients in each fold). At each iteration, one fold is selected as the testing dataset and the remaining nine folds are used as the training data for constructing the prediction model. The following process was repeated 10 times so that each fold would be used exactly once as the testing data: i) In the training set, we fit univariate Cox proportion hazard model for each gene and 21 genes most significantly associated with survival (gene were ordered by q-value from small to large and then filtered by q-value <0.01 after BH adjustment and absolute coefficient >1) were selected; ii) We then fit a multivariate Cox proportion hazard model with selected genes and computed a risk score as the summation of product of gene expression and its corresponding coefficient over all selected genes, for each patient in the training set. The tertiles of risk scores from the training data were determined; iii) We used the Cox regression coefficients estimated from training data to calculate risk score for patients in the testing data and the tertiles learnt from the training data were used to classify the patients into high, medium, and low risk clusters. The algorithm resulted in roughly equal size of each risk cluster (Low risk : Intermediate risk : High risk=33:40:45). The survival curves of the three predicted risk groups were generated and p-value from log-rank test to compare the survival difference was calculated. For Oncotype DX and BCI evaluation, we obtained the gene list to replace the Step (i) feature selection process. The remaining cross-validation and risk prediction in Step (ii) and (iii) are kept the same as the semi-supervised learning procedure. We chose to re-train the model and did not apply the models trained from previous datasets to facilitate a fair comparison.

**PCA analysis**

We did a principal component analysis of Agilent array gene expression data. Supplementary figure 1 shows the first two PC plot. Normal and tumor samples are well separated by first PC, while ER+ and ER- tumor samples are well separated by second PC. We also denote preM and postM by different color. But the first two PCs cannot separate preM from postM. We have checked the third PC cannot split preM and postM as well.
