# Supplementary material for: The molecular landscape of premenopausal breast cancer
Source: Breast Cancer Res. 2015 Aug 7;17:104. doi: 10.1186/s13058-015-0618-8 (PMC4531812; doi:10.1186/s13058-015-0618-8)
Supplement: Additional file 13: Figure S1. — Venn diagram representing datasets from The Cancer Genome Atlas (TCGA) and Molecular Taxonomy of Breast Cancer International Consortium (METABRIC). Figure S2. Principle component analysis (PCA) of (A) Agilent array and (B) methylation data. Figure S3. Differentially expressed (DE) genes between premenopausal (preM) and postmenopausal (postM) estrogen receptor-positive (ER+) tumors. Figure S4. Mutation spectra comparing somatic mutations identified in preM and postM ER+ tumors using MutSig. Figure S5. Differences in protein expression between preM and postM ER tumors (RPPA). Figure S6. Top canonical pathways enriched in preM ER+ tumors in TCGA RNA-Seq and TCGA Agilent. Figure S7. Top pathways identified in DAVID. Figure S8. Heatmap for top 50 entities in PARADIGM analysis when integrating Agilent array, copy number variation (CNV), somatic mutation and methylation data. Figure S9. Comparison of expression of laminin and integrin genes between preM and postM ER+ tumors. Figure S10. Hierarchical clustering of ER+ preM patients on the top 2,500 variable genes: (a) Agilent array; (b) RNA-Seq. Figure S11. LumA sub-cluster. (DOCX 3009 kb) [file 13058_2015_618_MOESM13_ESM.docx]

**Supplementary figures**


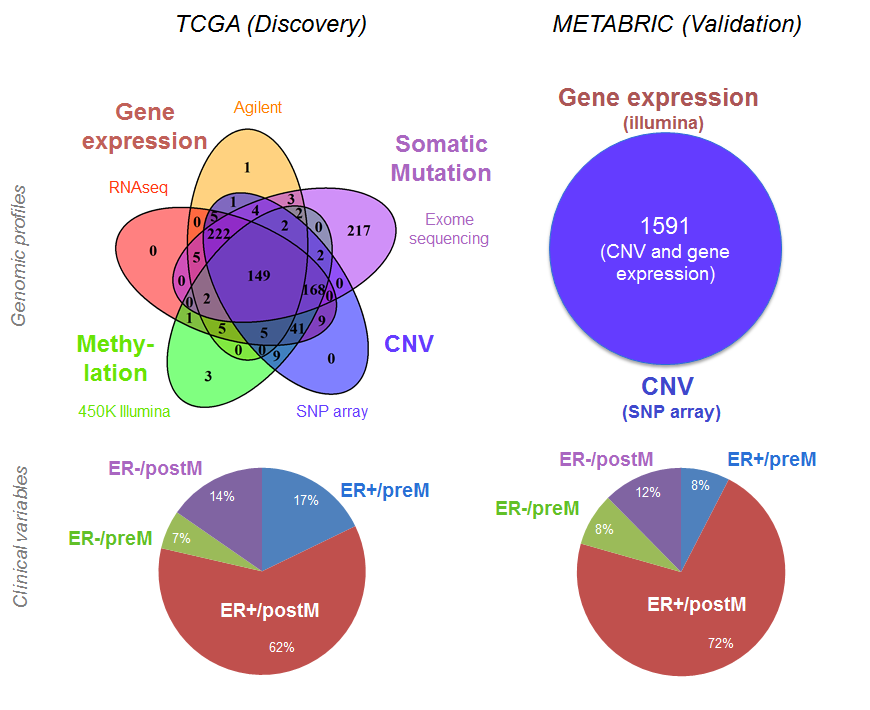


**Fig. S1 Venn diagram representing datasets from TCGA and METABRIC**: For analysis of TCGA data, we used data from gene expression, CNV, methylation, and somatic mutation. (In addition, we used RPPA data, not shown here). For analysis of METABRIC data, we used gene expression and CNV data for all 1591 tumor samples. The distribution of tumor samples with respect to ER and menopausal status is similar in both data sets: there are more ER+ tumor samples compared to ER- samples, and there is an enrichment of postM in ER+ samples compared with that in ER- samples. Survival information in TCGA is limited compared to information provided for the METABRIC data set.

1. **Agilent expression array**

**
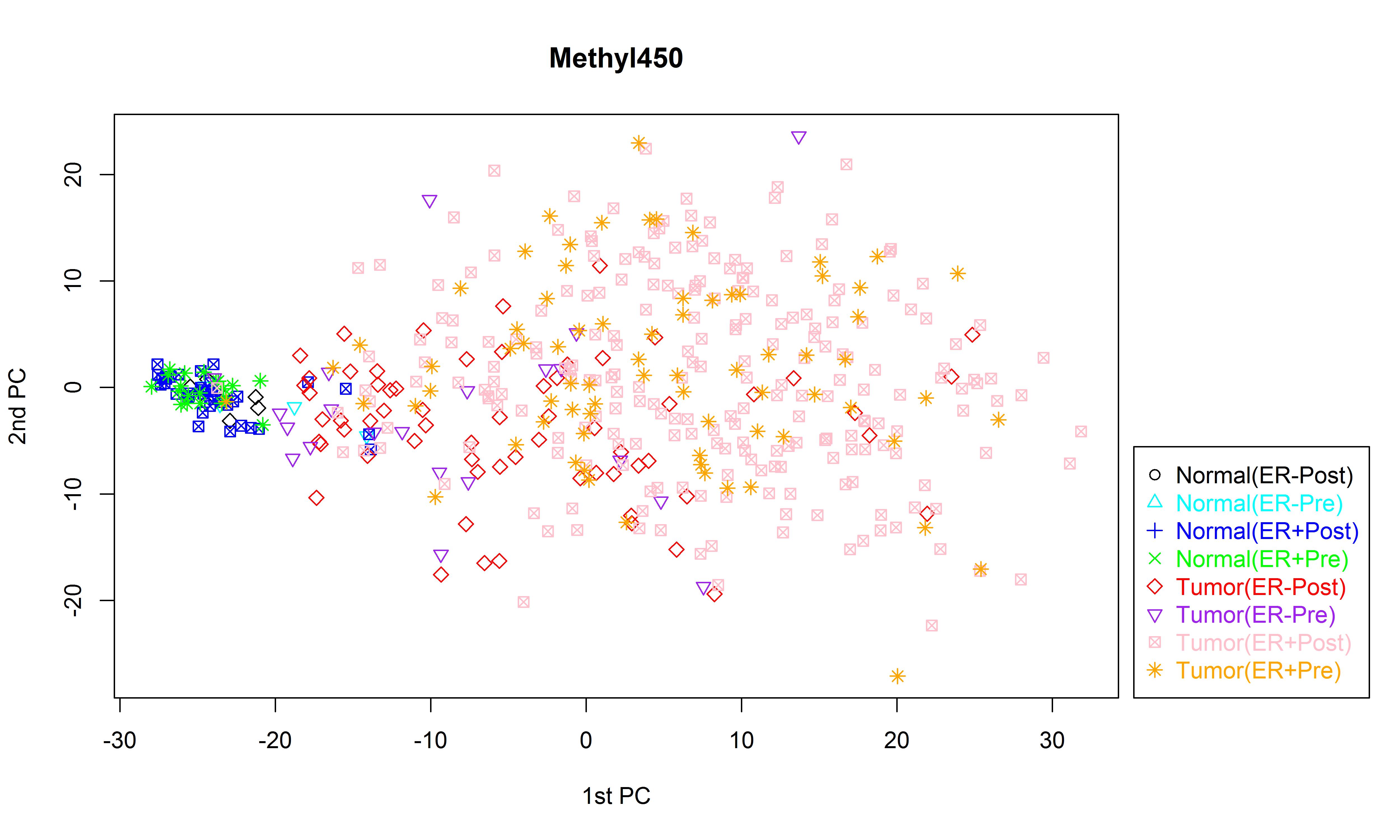
**

**(B) Methylation**

**Fig. S2: Principle component analysis (PCA) of A) Agilent array and B) methylation data.** The colors and symbols are defined as:

Tumor: purple triangle – ER-/preM; red diamond - ER-/postM; orange star – ER+/preM, pink square – ER+/postM

Normal: light blue triangle – ER-/preM; black diamond – ER-/postM; green star – ER+/preM; dark blue square – ER+/postM


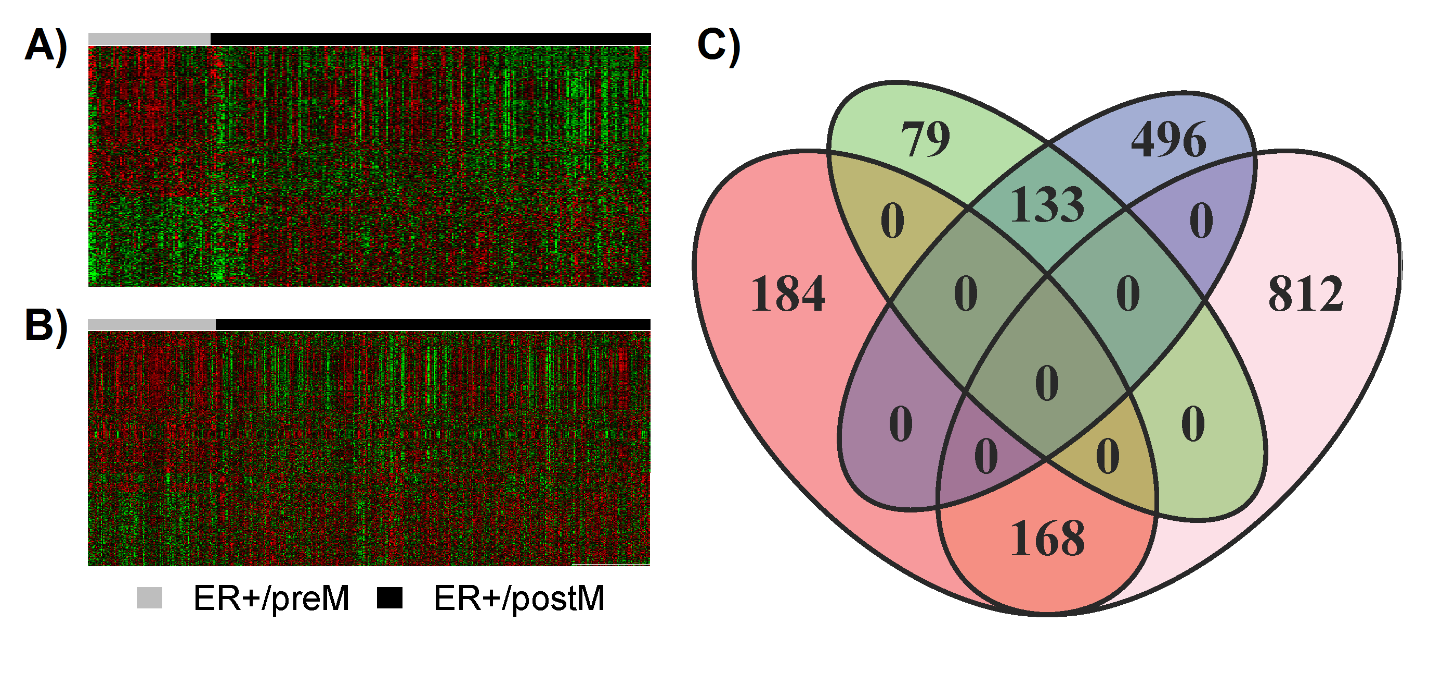


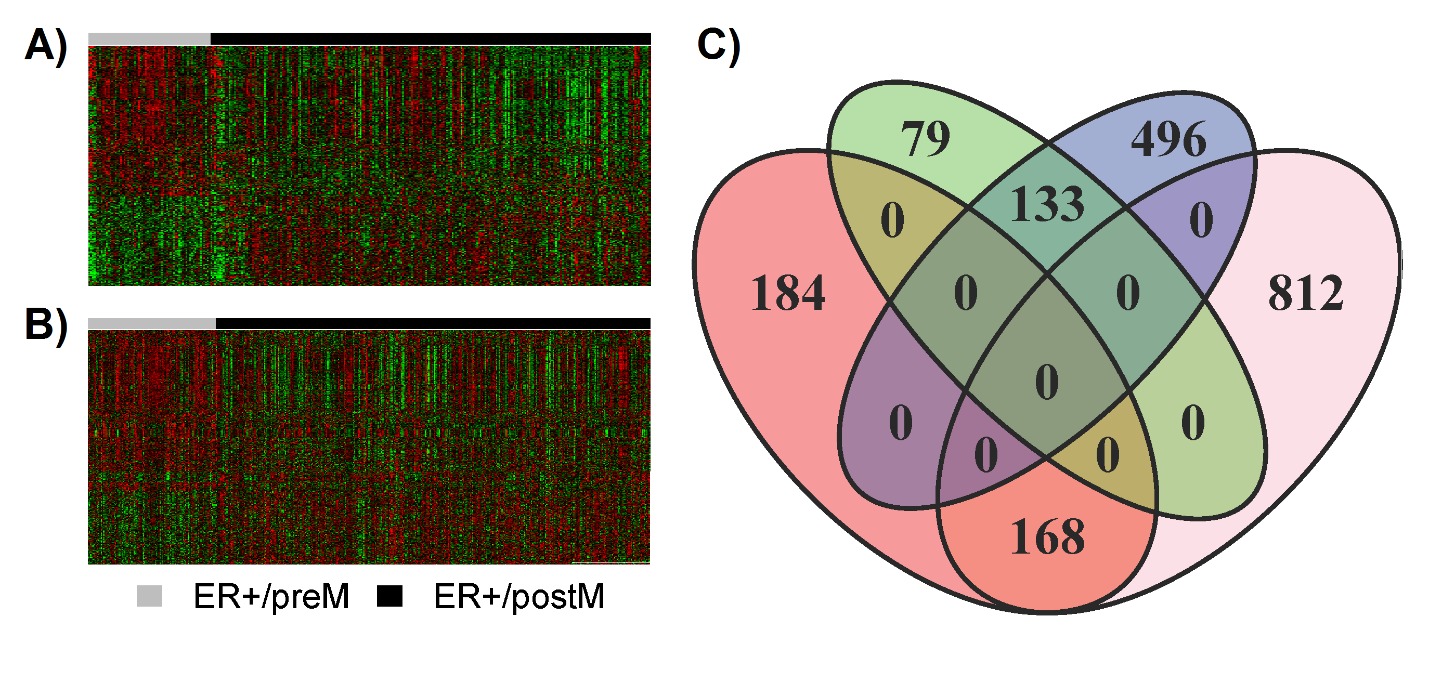
**B)**

**Fig S3: Differentially expressed (DE) genes between preM and postM ER+ tumors.** A) DE genes using Agilent microarray data. In the heatmap, each gene is normalized to standard normal distribution, and green and red indicate lower and higher expression, respectively. Grey bar – ER+/preM; black bar – ER+/postM. B) Venn Diagram of DE (ER+/preM vs. ER+/postM) from two different platforms: (1) Genes over-expressed in preM for Agilent (red); (2) Genes over-expressed in postM for Agilent (green); (3) Genes under-expressed in preM for Agilent (blue); (4) Genes under-expressed in postM for Agilent (pink). There are 168 and 133 genes overexpressed in postM and preM ER+ tumors, respectively, that overlap between the two platforms (without constraint of fold change).

**Fig. S4: Mutation spectra comparing somatic mutations identified in preM and postM ER+ tumors using MutSig.** A) Distribution of the six major classes of base pair mutations in pre and postM. B) Comparison of the percentage of transversions and transitions in pre and postM. C) Analysis of the specific trinucleotide content of base pair mutations in pre and postM. Note the increase in TCT>TAT and TCG>TTG in PostM. D) Analysis of C but only showing C>A and C>T conversions.

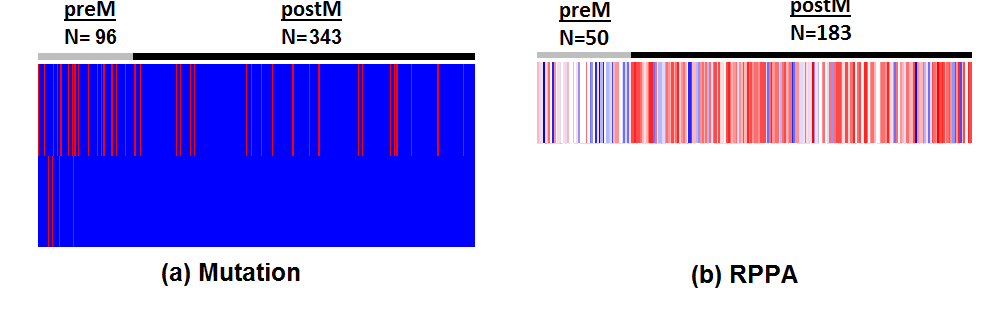


**Fig. S5: Differences in protein expression between preM and postM ER tumors.** RPPA: ER-alpha was detected to be statistically significant expressed between preM and postM ER+ tumors. Red and blue colors indicate higher and lower protein expression, respectively.

A)


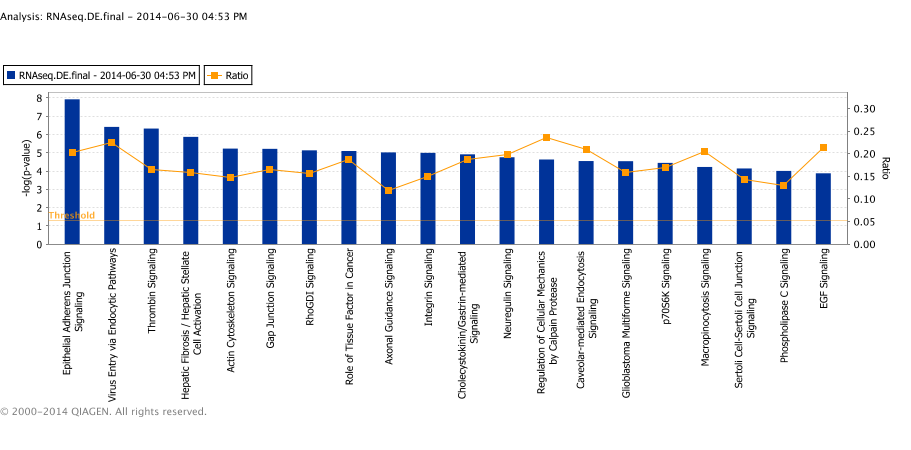


B)


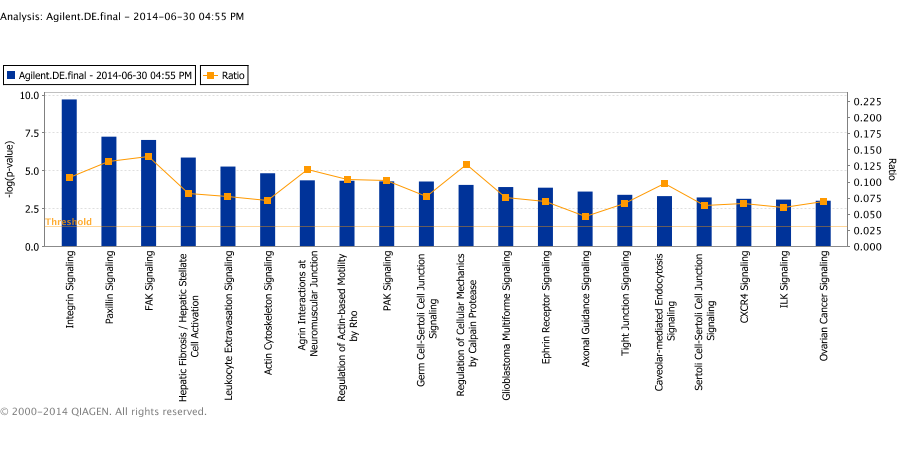


**Fig. S6 Top canonical pathways enriched in preM ER+ tumors in following datasets: (a) TCGA RNA-Seq (b) TCGA Agilent.**


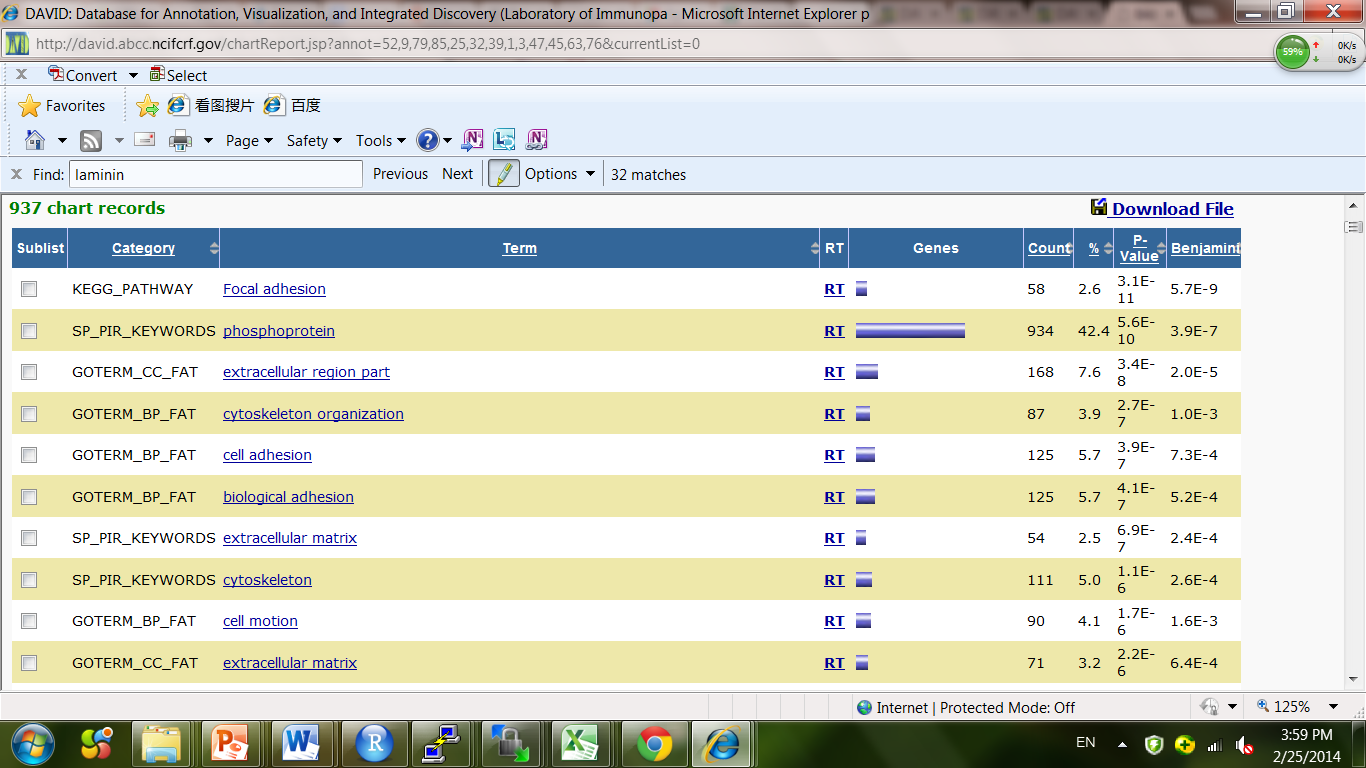


**Fig. S7 Top pathways identified in DAVID.**


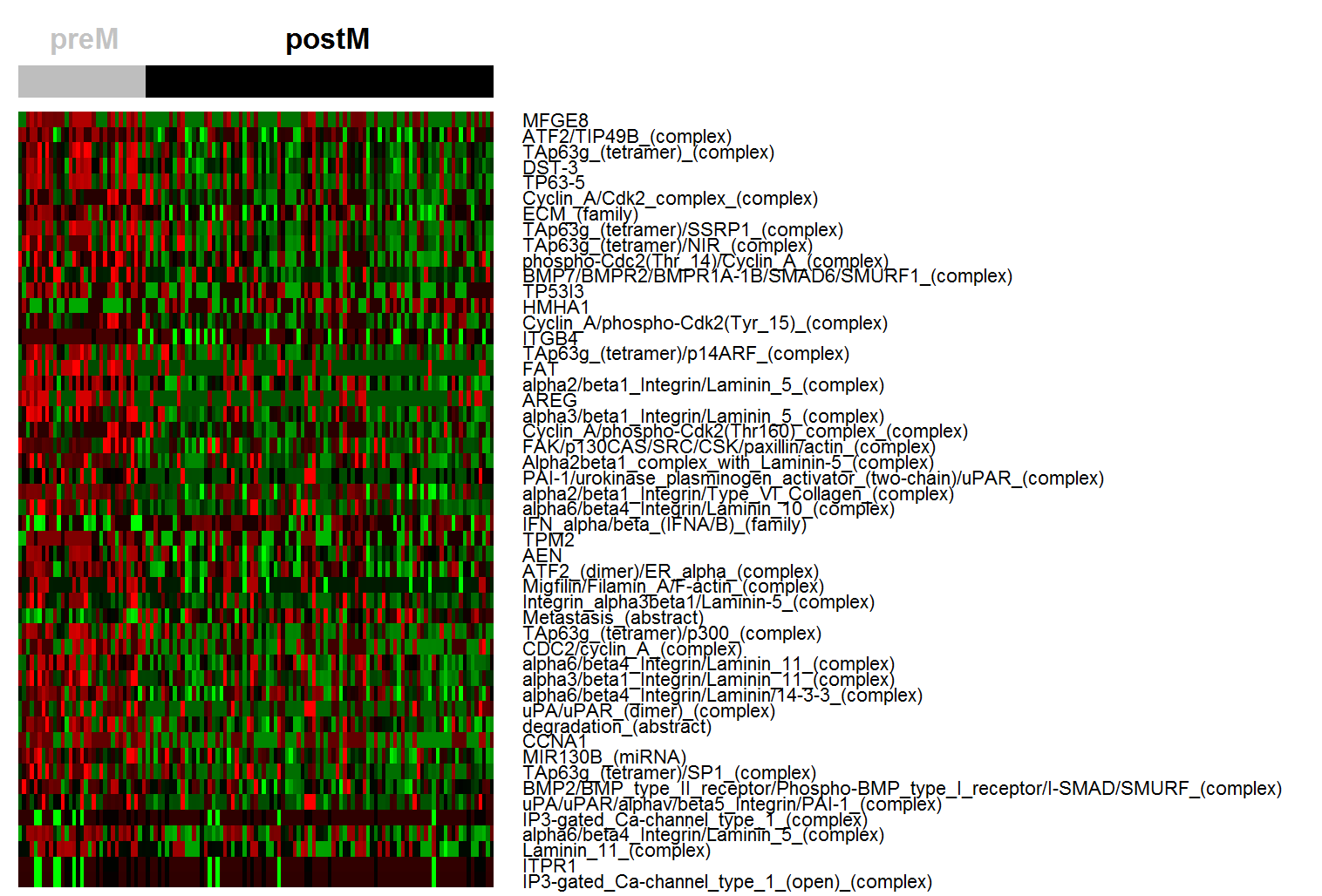


**Fig. S8 Heatmap for top 50 entities in PARADIGM analysis when integrating Agilent array, CNV, somatic mutation and methylation data.**


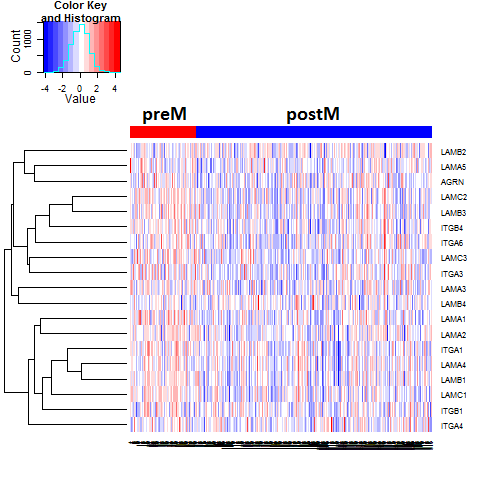


|  | preM(mean) | postM(mean) | fold.change | p.values |
| --- | --- | --- | --- | --- |
| LAMC1 | 0.967979439 | 0.747753782 | 1.294516272 | 7.73E-08 |
| LAMC2 | 3.245116153 | 2.075049423 | 1.56387415 | 1.57E-07 |
| LAMA1 | 0.283230535 | 0.224830918 | 1.259749046 | 1.43E-06 |
| LAMB1 | 0.704247126 | 0.567924855 | 1.240035755 | 2.69E-06 |
| LAMB3 | 1.540844487 | 0.911965399 | 1.689586566 | 1.65E-05 |
| ITGB4 | 2.162575307 | 1.647524375 | 1.31262113 | 2.57E-05 |
| ITGA1 | 1.30495943 | 1.040590832 | 1.254056243 | 0.000150298 |
| LAMC3 | 0.848193712 | 0.614841107 | 1.379533187 | 0.000185734 |
| LAMA2 | 22.11799623 | 17.42375365 | 1.269416262 | 0.00032031 |
| ITGA3 | 0.898205863 | 0.6863998 | 1.308575357 | 0.002071967 |
| LAMA3 | 3.268698896 | 2.830794249 | 1.154693209 | 0.007013705 |
| ITGA6 | 1.515482639 | 1.20984738 | 1.252622987 | 0.008158907 |
| AGRN | 1.619886663 | 1.380488258 | 1.173415749 | 0.013537334 |
| LAMA5 | 0.980504876 | 0.820097608 | 1.195595337 | 0.015973726 |
| LAMA4 | 1.453639938 | 1.336052332 | 1.088011228 | 0.021813721 |
| ITGB1 | 1.397262382 | 1.287032182 | 1.08564681 | 0.179564485 |
| ITGA4 | 0.310744831 | 0.303252299 | 1.024707254 | 0.424421192 |
| LAMB4 | 0.922431647 | 0.9694557 | 0.951494376 | 0.4724719 |
| LAMB2 | 2.377293848 | 2.470295332 | 0.962352079 | 0.887568482 |

**Fig. S9 Comparison of expression of laminin and integrin genes between preM and postM ER+ tumors.**


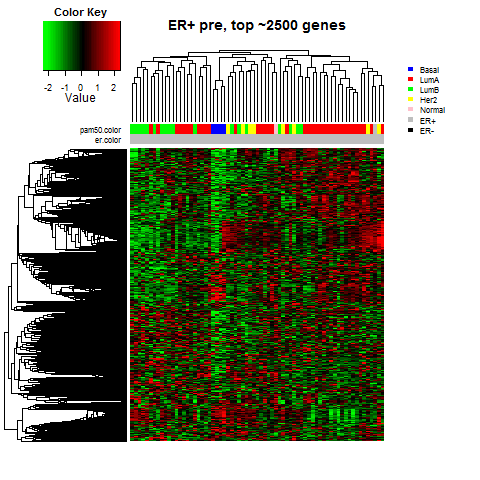

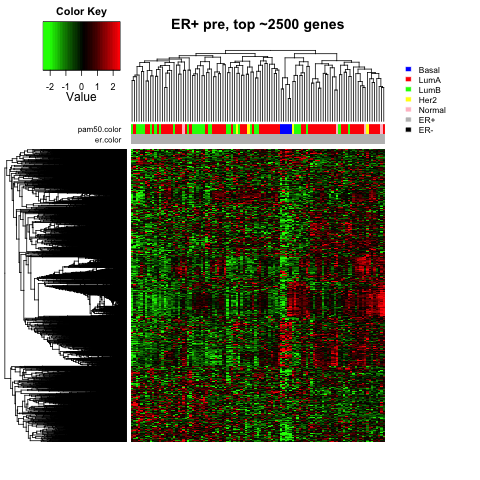


**Fig. S10 Hierarchical clustering of ER+ preM patients on top 2500 variable genes: (a) Agilent array; (b) RNA-Seq.**


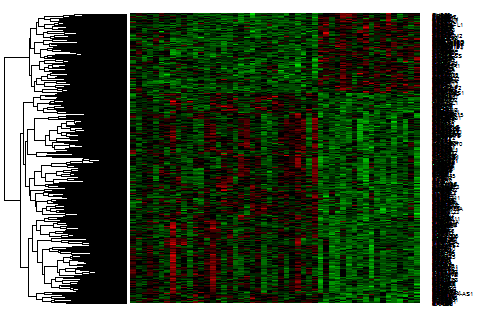

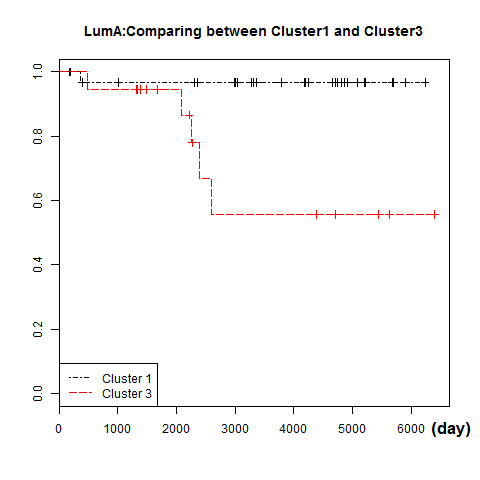


**Fig. S11 LumA sub-cluster.**
